# Hesperidin alleviates zinc-induced nephrotoxicity via the gut-kidney axis in swine

**DOI:** 10.3389/fcimb.2024.1390104

**Published:** 2024-04-29

**Authors:** Qingwen Yang, Lv Qian, Shanshan He, Chuanshi Zhang

**Affiliations:** Laboratory of Veterinary Pharmacology, Department of Animal Science and Technology, Chongqing Three Gorges Vocational College, Chongqing, China

**Keywords:** zinc, nephrotoxicity, hesperidin, gut-kidney axis, gut microbiota

## Abstract

**Introduction:**

Zinc (Zn) is an essential trace element in animals, but excessive intake can lead to renal toxicity damage. Thus, the exploration of effective natural antagonists to reduce the toxicity caused by Zn has become a major scientific problem.

**Methods:**

Here, we found that hesperidin could effectively alleviate the renal toxicity induced by Zn in pigs by using hematoxylin-eosin staining, transmission electron microscope, immunohistochemistry, fluorescence quantitative PCR, and microfloral DNA sequencing.

**Results:**

The results showed that hesperidin could effectively attenuate the pathological injury in kidney, and reduce autophagy and apoptosis induced by Zn, which evidenced by the downregulation of LC3, ATG5, Bak1, Bax, Caspase-3 and upregulation of p62 and Bcl2. Additionally, hesperidin could reverse colon injury and the decrease of ZO-1 protein expression. Interestingly, hesperidin restored the intestinal flora structure disturbed by Zn, and significantly reduced the abundance of Tenericutes (phylum level) and Christensenella (genus level).

**Discussion:**

Thus, altered intestinal flora and intestinal barrier function constitute the gut-kidney axis, which is involved in hesperidin alleviating Zn-induced nephrotoxicity. Our study provides theoretical basis and practical significance of hesperidin for the prevention and treatment of Zn-induced nephrotoxicity through gut-kidney axis.

## 1 Introduction

Zinc (Zn) is an essential trace element, which is involved in many important physiological processes (Hutchens et al., 2021). In eukaryotes, Zn plays an important role in biological processes such as energy metabolism, oxidative stress and signal transduction (Kim and Lee, 2021). A number of studies have shown that appropriate amount of Zn added to feed can improve the growth efficiency of animals (Li et al., 2021c). However, excessive intake of Zn can cause acute and chronic poisoning in animals (Kataba et al., 2021). Studies have pointed out that the toxic effect of Zn is mainly through disrupting the physiological homeostasis of lipid bimolecular structure and changing the structure of glycoprotein on the cell surface, and causing disorders to the structure and function of Zn-containing enzymes such as ATP and nucleotidase, resulting in reduction of ATP synthesis, dysfunction of membrane active transport, organelle edema and other diseases (Alhasawi et al., 2014; Ren et al., 2017). When animals are supplemented with excessive Zn, they will show loss of appetite, sluggish activity, diarrhea, growth and development arrest and other toxic symptoms. Recently, lots of studies have shown that the homeostasis of Zn is related to the occurrence and development of various diseases (Wang et al., 2020).

Hesperidin is a derivative of dihydroflavone and mainly exists in the peel of citrus fruits (Xiong et al., 2019). In recent years, hesperidin has attracted extensive attention in livestock and poultry breeding because of its antioxidant, lipid metabolism regulation, anti-inflammatory and other biological activities (Hager-Theodorides et al., 2021). It has been reported that hesperidin can effectively relieve the symptoms of heat stress in broilers and improve the growth efficiency of the animals, so it is gradually recommended to be used in animal feed as a feed additive (Kamboh et al., 2013; Kouvedaki et al., 2024). Studies have found that hesperidin regulates gut flora to alleviate liver damage in mice (Li et al., 2022). However, there are few studies on the mechanism of hesperidin in toxic diseases and in the regulation of intestinal flora.

The “gut microbiota-gut-kidney axis” theory was first proposed to explain how changes in gut microecology affect chronic kidney disease through regulation of metabolites (Nouri et al., 2022). Recent studies have found that a decrease in the abundance of intestinal probiotics in animal models of chronic kidney disease, accompanied by an increase in the abundance of pathogenic bacteria, which causes dyshomeostasis of intestinal flora, directly destroys intestinal barrier function and leads to bacterial translocation and the accumulation of enterogenous uremic toxins in the blood, thereby activating renal inflammation, oxidative stress and fibrosis pathways (Plata et al., 2019; Li et al., 2023a). In addition, excessive metabolic waste cannot be fully excreted by the kidney and re-enters the intestine, further aggravating the imbalance of intestinal flora and leading to the sustainable development of the disease. Therefore, the discovery or development of effective drugs through the “gut microbiota - enteric-kidney axis” is of great significance for the treatment of kidney diseases (Hsu and Tain, 2022). At present, there are still gaps in the research on the role and mechanism of “gut - kidney axis” in natural compounds on chronic kidney injury caused by heavy metals in animals, which is also the focus of the scientific community in recent years.

Studies have confirmed that high levels of Zn can induce kidney damage (Ratn et al., 2018). At the same time, hesperidin has been shown to have significant effects in maintaining intestinal flora homeostasis and alleviating kidney injury, but its underlying mechanism is still unclear (Mas-Capdevila et al., 2020). In this study, pigs were used as experimental animals, and hesperidin was used for treatment based on the establishment of a pathological injury model of high levels of Zn. Based on the theory of “intestinal microbiota - enteric-kidney axis”, changes in intestinal flora structure and metabolites, changes in intestinal barrier function, renal function, and dynamic processes of autophagy and apoptosis were detected by detecting changes in intestinal flora of pigs. Further, the mechanism of “intestinal flora - intestinal renal axis” in hesperidin alleviating kidney injury caused by Zn poisoning was discussed, which laid a foundation for elucidation of the protective mechanism of hesperidin in regulating kidney injury of pigs with high levels of Zn.

## 2 Materials and methods

### 2.1 Animal treatment

A total of 80 weaned pigs, one-month-old (approximate 10 kg) were fed with basal mixed ration for one week during adaptive phase (**Supplementary Table S1**). Pigs were randomly divided into four groups: control (75 mg/kg anhydrous Zn sulfate), Zn group (1500 mg/kg anhydrous Zn sulfate), Zn+hes group (1500 mg/kg anhydrous Zn sulfate + 150 mg/kg hesperidin), and hes group (150 mg/kg hesperidin). The feed was supplemented with anhydrous Zn sulfate and/or hesperidin, and the periods lasted for 40 days. The samples were collected under anesthesia (sodium pentobarbital) on day 40. The experiment was approved from the Animal Care and Use Committee of Chongqing Three Gorges Vocational College.

### 2.2 Hematoxylin-eosin staining

After deparaffinization and rehydration of the tissue sections, they were stained with hematoxylin and eosin, followed by dehydration with gradient alcohol and transparency with xylene, and finally sealed with neutral resin.

### 2.3 Ultrastructure observation

The kidney tissues were collected and incubated in osmium tetroxide for 2 h. Then, the samples were performed as previous study (Fang et al., 2021). The ultrathin sections were detected by a HITACHI HT 7800 transmission electron microscope (HITACHI, Japan).

### 2.4 Immunohistochemical observation

The section preparation, antigen restore, peroxidase clearance and blocking in immunohistochemistry were carried out according to the previous studies (Liao et al., 2020). The primary antibody (ZO-1) was incubated for 16 h, followed by incubation with the secondary antibody conjugated with biotin for 1 h. The slides were observed and photographed under a microscope (Leica, Germany).

### 2.5 mRNA expression levels analysis

Total RNA was isolated with Trizol reagent (Takara, Japan). Reverse transcription steps of cDNA were carried out by using BeyoRT™ II cDNA synthesis kit (Beyotime, China). The primers of LC3, p62, ATG5, Bak1, Bax, Bcl2, Caspase-3 and GAPDH were presented in **Supplementary Table S2**. The RT-qPCR was operated on the Applied Biosystems SimpliAmp PCR System (Thermo Fisher, USA). The results of relative mRNA expressions were shown as 2^-△△CT^.

### 2.6 Microfloral DNA extraction and sequencing

Intestine flora DNA was collected by HiPure Stool DNA Kits (Magen, Guangzhou, China) following the producer’s instructions. A set of V3/V4 conserved region was amplified by PCR. The amplified PCR products were extracted by using DNA Gel Extraction Kit (Beyotime, China) for target fragment recovery. Purified amplicons were sequenced according to previous study (Xie et al., 2022). Prediction of flora function was determined on the TaxFun platform (Gene Denovo, China).

### 2.7 Statistical analysis

GraphPad Prism 8.5 (GraphPad Inc., LaJolla, CA, USA) was used for data statistics. The collected data were expressed as mean ± standard errors and analyzed by one-way analysis of variance (ANOVA). The statistical significance was deemed at *p* < 0.05.

## 3 Results

### 3.1 Effects of Zn on the growth performance and renal injury

**Figure 1:**
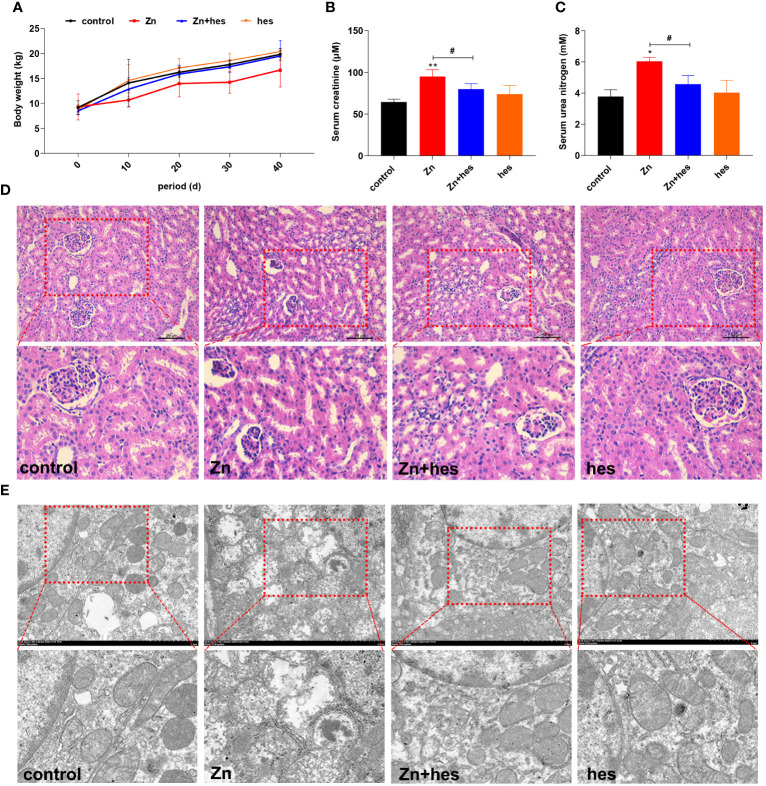
Effects of Zn on the growth performance and renal injury in pigs. **(A)** Body weight. **(B)** Serum creatinine. **(C)** Urea nitrogen. **(D)** HE staining. **(E)** TEM observation.

As shown in **Figure 1A**, the body weight of pigs in Zn group was decreased compared to control group. Meanwhile, the levels of creatinine and urea nitrogen in serum were remarkably increased under Zn treatment compared to control group (*p*<0.05 or *p*<0.01). Hesperidin increased body weight and significantly decreased creatinine and urea nitrogen levels (*p*<0.05) (**Figures 1B, C**). Histological observations showed that excessive exposure to Zn caused significant glomerular atrophy compared with control group. However, after hesperidin treatment, the pathological changes were significantly improved (**Figure 1D**). Through further ultrastructural observation, we found that mitochondria in the kidney exposed to high level of Zn showed vacuolation and ridge breakage, while hesperidin combined with Zn treatment group did not show a large number of damaged mitochondria (**Figure 1E**).

### 3.2 Effects of hesperidin on Zn-induced autophagy and apoptosis in kidney

**Figure 2:**
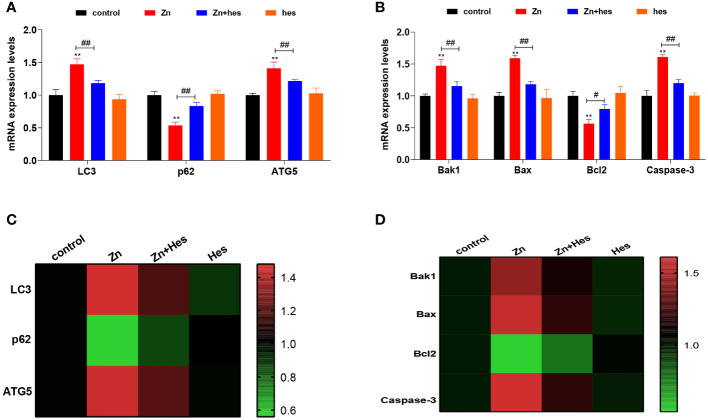
Effects of Zn on the autophagy and apoptosis in kidney. **(A)** mRNA levels of LC3, p62, and ATG5. **(B)** mRNA levels of Bak1, Bax, Bcl2, and Caspase-3. **(C)** Heat mat of the autophagy-related genes expression. **(D)** Heat mat of the apoptosis-related genes expression. “*” expressed the statistical difference compared with the control group (*P < 0.05, **P < 0.01 and ***P < 0.001). “#” expressed the statistical difference between the two groups (#P < 0.05, ##P < 0.01 and ###P < 0.001).

As shown as **Figure 2**, the mRNA levels of LC3, ATG5, Bak1, Bax, and Caspase-3 were significantly upregulated in Zn group compared to control group (*p*<0.01), and the mRNA expression levels of p62 and Bcl2 were markedly downregulated (*p*<0.01). Additionally, hesperidin could remarkably reduce the mRNA levels of LC3, ATG5, Bak1, Bax, and Caspase-3 and elevate the levels of p62 and Bcl2 under Zn treatment.

### 3.3 Effects of hesperidin on Zn-induced colonic barrier dysfunction

**Figure 3:**
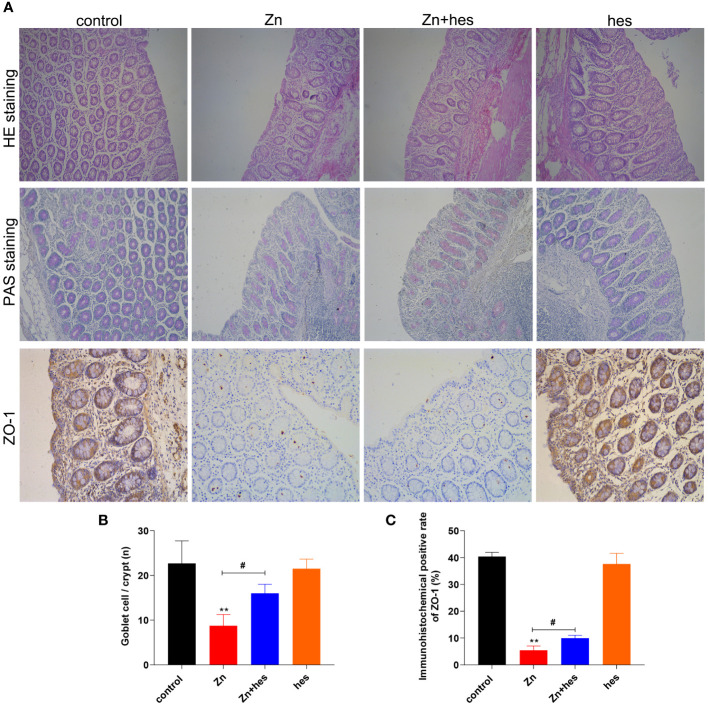
Effects of Zn on the barrier function in colon. **(A)** HE staining, PAS staining, and immunofluorescencal detection of ZO-1 in colon. **(B)** The number of goblet cells. **(C)** Immunofluorescence positive rate of ZO-1. “*” expressed the statistical difference compared with the control group (*P < 0.05, **P < 0.01 and ***P < 0.001). “#” expressed the statistical difference between the two groups (#P < 0.05, ##P < 0.01 and ###P < 0.001).

Here, after excessive Zn intake, the colon mucosa is damaged and the number of glands is significantly reduced (**Figure 3A**), and the PAS staining also showed that the number of goblet cells was decreased significantly under Zn treatment in contrast to the control group (*p*<0.05) (**Figures 3A, B**). Whereas, hesperidin could signally relieve the pathological damage of the colon (**Figures 3A, B**). In addition, the result of immunohistochemistry revealed that the tight junction protein ZO-1 was expressed at a low level under excess Zn treatment (*p*<0.01), and hesperidin can significantly reverse the trend (*p*<0.05) (**Figures 3A, C**).

### 3.4 Effects of hesperidin on alpha and beta diversity indices in intestinal microflora under Zn treatment

**Figure 4:**
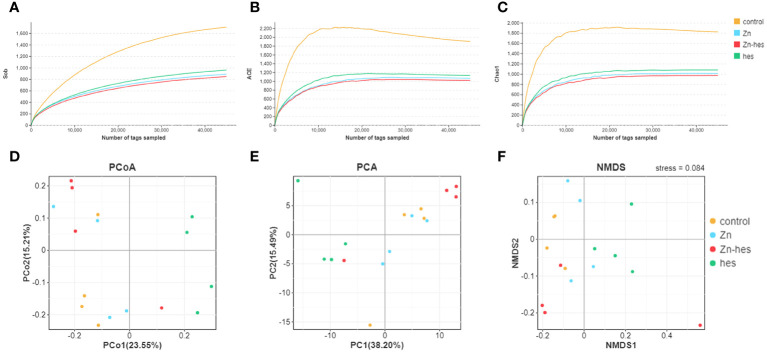
Effects of Zn on alpha and beta diversity indices in colonic microflora. **(A–C)** Alpha diversity is analyzed by Sob **(A)**, ACE **(B)**, and chao1 **(C)**. **(D–F)** Beta diversity is analyzed by PCoA **(D)**, PCA **(E)**, and NMDS **(F)**. Each dot represents an individual sample.

The alpha diversity of gut flora was analyzed by the Sob, ACE, and chao1. Here, the Sob (**Figure 4A**), ACE (**Figure 4B**), and chao1 (**Figure 4C**) in the control group were higher than that in Zn group, hes group, and Zn+hes group. Additionally, PCoA, PCA, and NMDS plot were used to evaluate Beta-diversity. Different colored spots represented the different groups. The gathered spots meant that the composition of the microbial structure between samples is more similar. In this study, the PCoA, PCA plot, and NMDS showed that each group’s plots were independent of the other group (**Figures 4D–F**).

### 3.5 Effects of hesperidin on the composition of the gut microbiota under Zn treatment

**Figure 5:**
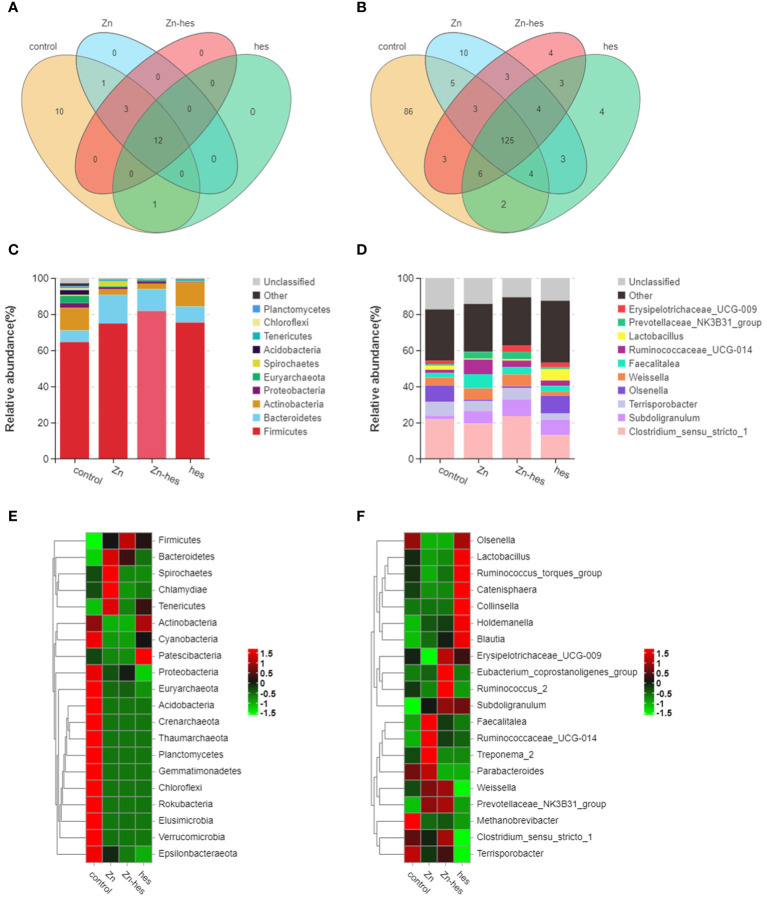
Effects of Zn on the changes in intestinal flora. **(A)** Venn diagram of gut flora at the phylum level. **(B)** Venn diagram of gut flora at the genus level. **(C)** Composition of gut flora at the phylum level. **(D)** Composition of gut flora at the genus level. **(E)** Heat map of gut flora at the phylum level. **(F)** Heat map of gut flora at the genus level.

**Figure 6:**
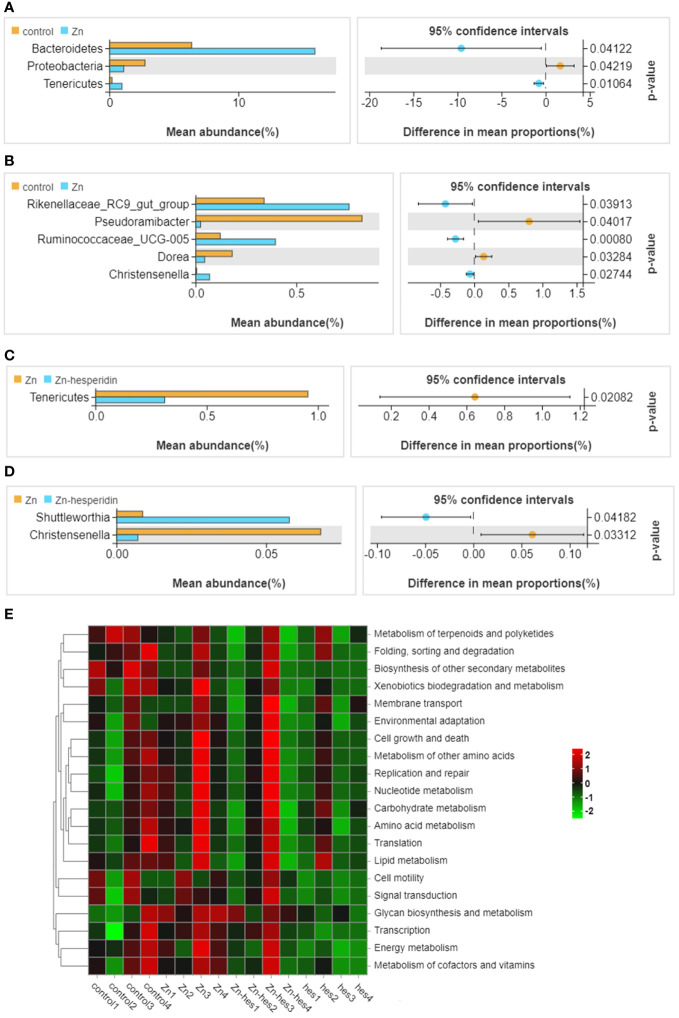
Differential flora screening and functional prediction. **(A)** Different bacteria at the phylum level between control group and Zn group. **(B)** Different bacteria at the genus level between control group and Zn group. **(C)** Different bacteria at the phylum level between Zn group and Zn+hes group. **(D)** Different bacteria at the genus level between Zn group and Zn+hes group. **(E)** Function prediction of differential flora.

In the present study, the results of venn diagram showed at the phylum level, a total of 16 OTUs in the control group and the Zn group, and a total of 15 OTUs both in Zn group and Zn+hes group. At the level of genus, a total of 137 OTUs in the control group and the Zn group, and a total of 135 OTUs both in Zn group and Zn+hes group (**Figures 5A, B**). We found that the Firmicutes is the dominant flora. Compared with the control group, the abundance of Bacteroidetes was increased and Actinobacteria was decreased in the Zn group, while the Zn+hes group showed a reverse trend compared with the Zn group, and the hes group and the control group were close at phylum level (**Figures 5C, E**). In addition, the abundance of Ruminococcaceae_UCG-014 was increased and Olsenella and Lactobacillus were decreased in the Zn group, while the Zn+hes group showed a reverse trend compared with the Zn group at genus level (**Figures 5D, F**). Through the significance analysis, it was found that the abundance of Bacteroidetes and Tenericutes were increased significantly and Proteobacteria was decreased remarkably in Zn group compared to control group at phylum level. Interestingly, hesperidin could markedly decrease the abundance of Tenericutes under Zn treatment (**Figures 6A, C**). At genus level, the abundance of Rikenellaceae_RC9_gut_group, Ruminococcaceae_UCG-005, and Christensenella were increased remarkably in Zn group compared to control group, and Pseudoramibacter and Dorea were significantly decreased. Meanwhile, hesperidin could decrease the abundance of Christensenella under Zn treatment (**Figures 6B, D**). Besides that, the function prediction possibly indicated that the differential flora might participate in the cell growth and death, nutrition metabolism, energy metabolism and so on (**Figure 6E**).

## 4 Discussion

Zn is an essential trace element involved in a variety of life processes. Dietary supplementation of appropriate amount of Zn can not only improve animal reproductive performance and maintain intestinal microenvironment homeostasis, but also improve antioxidant function and enhance immunity. However, excessive intake of Zn in animals can lead to decreased antioxidant function, resulting in oxidative stress and programmed death (Yang et al., 2022; Li et al., 2023b). It has been proved that the kidney is an important target organ for Zn toxicity, but its pathogenic mechanism is still unclear. The “gut-kidney axis” theory holds that the disturbance of intestinal flora can induce the impairment of intestinal barrier function and systemic micro-inflammatory response, thus inducing the functional impairment of the kidney (Yang et al., 2018). Therefore, through the search for corresponding drugs, through the “entero-renal axis” to treat kidney disease has become a hot research direction. Hesperidin is a kind of natural flavonoid widely found in citrus fruits, which has been found to have antioxidant, anti-inflammatory and other biological activities (Li et al., 2021b). Here, this study identified the mechanism of “intestinal flora - intestinal kidney axis” in hesperidin alleviating kidney injury caused by Zn, and further clarified the pathway of hesperidin in Zn-induced kidney injury.

A large number of studies have confirmed that cell damage caused by heavy metals is often accompanied by autophagy and apoptosis (Fang et al., 2021; Li et al., 2021a). Autophagy is a biological mechanism widely existing in eukaryotic cells, and it is considered to be an important way for cells to degrade large quantities of senescent proteins and damaged organelles. Autophagy forms an independent double-layer membrane structure, wraps the cytoplasm under the regulation of a variety of proteins, fuses with lysosomes and finally degrades into small molecules, which are released back into the cytoplasm for use (Klionsky et al., 2021). During autophagy, ATG5 promotes the formation of bilayer membrane of autophagosome, recruits LC3 and promotes its transformation from LC3-I to LC3-II. The degradation of p62 is another marker for monitoring autophagy, as p62 can bind to LC3 and be selectively degraded by autophagy (Levine and Kroemer, 2019). In addition, apoptosis is a type of programmed cell death that is highly regulated by multiple genes and characterized by chromatin condensation, DNA cleavage, and the formation of apoptotic bodies. The mechanism of apoptosis is very complex, mainly divided into endogenous and exogenous pathways (D'Arcy, 2019). Among them, most of the research focuses on endogenous apoptosis, and the apoptosis of mitochondrial pathway has also become a research hotspot for most scholars. In most cases, mitochondria-mediated endogenous apoptosis is controlled by the Bcl family, which is composed of pro-apoptotic factors such as Bax and Bak-1 and anti-apoptotic factors Bcl-2 (Yuan et al., 2021). When the apoptotic signal is received, the pro-apoptotic protein will be transferred to the mitochondrial membrane, and the Bcl-2 protein will be further down-regulated, resulting in the permeability of the mitochondrial outer membrane, the reduction of mitochondrial membrane potential, and the release of intermembrane pro-apoptotic substances, which will further promote the shear of Caspase-3, destroy the nuclear structure, and break down the cell into apoptotic bodies (Liao et al., 2019). It has been confirmed that feeding high dose of copper can increase the protein expression levels of LC3-II/LC3-I, ATG5, Bax, and cleaved Caspase-3 (Liao et al., 2020, 2021). Zhang et al. also confirmed that molybdenum and cadmium combined feeding in ducks also led to renal autophagy and apoptosis (Zhang et al., 2022b). In our study, we have found that Zn could induced the high expression level of autophagy and apoptosis-related genes, and hesperidin could reverse these effects, which verified the anti-renal damage effect of hesperidin.

Studies have shown that the toxic effect of heavy metals is caused by the absorption of digestive tract and metabolism in the body (Witkowska et al., 2021). At the same time, intestinal damage may become an important bridge for toxin-induced organ toxicity damage (Liao et al., 2022). Therefore, the evaluation of intestinal damage is also an important indicator for heavy metal poisoning. In this study, we evaluated the intestinal toxicity of Zn by histology and expression of ZO-1 protein. ZO-1 is one of intestinal tight junction proteins, which can maintain the structural integrity of intestinal epithelial cells and the continuity of intestinal mucosal barrier, and plays an important role in cell proliferation, differentiation and growth, regulation of intercellular signal transduction and penetration (Kuo et al., 2022). Here, we found that Zn exposure could cause pathological damage to the colon, and induce the ZO-1 protein expression decreased significantly. Nevertheless, hesperidin treatment could upregulate the ZO-1 expression level, which indicating that hesperidin can repair barrier barriers, improve the structure and function of intestinal epithelium, and regulate intestinal mucosal permeability.

In this study, we have revealed that Zn can cause nephrotoxicity, and the colon also produces pathological damage, and hesperidin can reverse the damage both of them. It has reported that the process of drug alleviating kidney injury may involve the role of the “gut-kidney” axis, especially the function of intestinal flora (Xie et al., 2022). Therefore, we speculate that hesperidin plays a key role in alleviating Zn-induced nephrotoxicity by the gut microbiota. In recent years, with the attention paid to the occurrence of kidney diseases caused by intestinal microecological disorders, the relationship between gut and kidney has gradually become a hot topic of research, and the theory of “gut-kidney axis” has been gradually confirmed (Evenepoel et al., 2017). As reported, 20% of patients with inflammatory bowel disease have mild tubular damage, which may be related to the effects of conventional treatment drugs and inflammatory cytokines. The proposal of the “gut-kidney” axis explores the regulating effect of the gut on the kidney. As an important role of the “gut-kidney” axis, intestinal flora plays an important role, and the disturbance of gut microbiota homeostasis and the intestinal barrier dysfunction are the inducing factors in the occurrence of kidney diseases (Chen et al., 2019). Here, we found that Zn could remarkably increase the abundance of Tenericutes and Christensenella, and hesperidin significantly decrease their abundance. Zhang et al. showed that the Tenericutes was reduced in diabetic nephropathy after Lycoperoside H treatment, which provided an important reference for the involvement of Tenericutes in the regulation of kidney diseases through “gut-kidney” axis (Zhang et al., 2022a). In addition, as a probiotic, Christensenella has been used for the treatment of diabetes, which could enhance the intestinal barrier and reduce intestinal inflammation (Pan et al., 2022). Therefore, hesperidin was found to improve Zn-induced intestinal barrier dysfunction by changing the abundance of Tenericutes and Christensenella, thus alleviating zinc-induced kidney injury via “gut-kidney” axis. In subsequent studies, we can use high-throughput sequencing to screen signal molecules to explore the molecular targets of hesperidin in regulating zinc-induced kidney injury.

## 5 Conclusion

In summary, Zn could induce nephrotoxicity and intestinal damage, and the disordered intestinal flora, as the core of “gut-kidney” axis, is an important pathway to promote Zn-induced nephrotoxicity. Additionally, hesperidin could improve the zinc-induced gut microbiota disorder for alleviating Zn-induced nephrotoxicity *via* “gut-kidney” axis.

## Data availability statement

The raw data supporting the conclusions of this article will be made available by the authors, without undue reservation.

## Ethics statement

The animal studies were approved by South China Agricultural University ethics committee. The studies were conducted in accordance with the local legislation and institutional requirements. Written informed consent was obtained from the owners for the participation of their animals in this study.

## Author contributions

QY: Writing – original draft. LQ: Writing – review & editing. SH: Writing – review & editing. CZ: Writing – review & editing.
