# Supplementary material for: Hesperidin alleviates zinc-induced nephrotoxicity via the gut-kidney axis in swine
Source: Front Cell Infect Microbiol. 2024 Apr 29;14:1390104. doi: 10.3389/fcimb.2024.1390104 (PMC11089138; doi:10.3389/fcimb.2024.1390104)
Supplement: Supplementary file 1 [file Table_1.docx]

**Supplementary materials**

**Table S1**. The ingredient and nutritional composition in the basic diet

| Ingredient | % | Nutritional composition |  |
| --- | --- | --- | --- |
| Corn | 65.60 | Digestible energy (MJ/kg) | 13.75 |
| Bean flour | 22.00 | Crude protein (%) | 17.85 |
| Wheat bran | 11.00 | Lysine (%) | 0.95 |
| Coarse fiber | 9.00 | Methionine +cystine (%) | 0.38 |
| Granulesten | 1.50 | Calciuml (%) | 0.84 |
| Fish flour | 3.00 | Phosphorus (%) | 0.63 |
| Meat flour | 1.00 |  |  |
| Salt | 0.40 |  |  |
| Limestone | 0.80 |  |  |
| Dicalcium phosphate | 0.80 |  |  |
| Bone meal | 1.00 |  |  |
| Additive[*](https://www.sciencedirect.com/science/article/pii/S0946672X07001435#tblfn1b) | 0.45 |  |  |

* Per kilogram of diet: Cu (10 mg); Fe (150 mg); Zn (200 mg); Mn (10 mg); I (0.15 mg); Se (0.30 mg); vitamin A (4400 IU); vitamin D (440 IU); vitamin E (22 IU); vitamin K (1.1 mg); d-pantothenic acid (22 mg); niacin (22 mg); vitamin B_12_ (22 mg); choline (0.61 g); d-biotin (0.14 mg) and folic acid (0.66 mg).

**Table S2**. Primer sequences for qPCR.

| Gene | Sense sequences (5´-3´) | Antisense sequences (5´-3´) |
| --- | --- | --- |
| Atg5 | GCCATCAATCGGAAACTCAT | TGAAGCCACAGGACGAAAG |
| LC3 | TGCAGCTCAATGCTAACCAA | CTTCATCCTTCTCGCTTTCG |
| P62 | AGCTGCCCTCAGCCCTCTCTA | GGCTTCTCTTCCCTCCATG |
| Bax | AAGCGCATTGGAGATGAACT | GGCCTTGAGCACCAGTTTAC |
| Bak1 | ATGACATCAACCGGCGATAC | TTGATGCCACTCTCGAACAG |
| Bcl2 | CGGCGATGACTTCTCTCGT | TTGACGCTCTCCACACACAT |
| Caspase-3 | GCAGTTTTATTTGCGTGCTTC | TCCGTCTCAATCCCACAGTC |
| GAPDH | ACCCAGAAGACTGTGGATGG | AAGCAGGGATGATGTTCTGG |
